# Supplementary material for: A goat experimental epistaxis model: Hemostatic effect of stop nosebleeds device
Source: PLoS One. 2025 Jun 10;20(6):e0324727. doi: 10.1371/journal.pone.0324727 (PMC12151359; doi:10.1371/journal.pone.0324727)
Supplement: S1 File — (PDF) [file pone.0324727.s001.pdf]

KING F[illegible]AL SPECIALIST HOSPITAL AND RESEARCH [illegible]NTRE

COMPARATIVE MEDICINE DEPARTMENT

LABORATORY ANIMAL SERVICES

Surgery Record

**RAC Protocol #:** 2230013	**Animal ID#:** C093364 (01)	**Microchip#:**		
**Principal Investigator:** DR. Falath	**Contact #:**	**Species:** OVINE/SHEEP ♂		
**Surgeon:**	**Contact #:**	**Pre-Surgical Evaluation:**		
**Surgical Procedure:** A Sheep (Ovis aries) Experimental Epistaxis model: Hemostatic Effect of Stop Nosebleeds Device	**DATE:** 6/3/24	**BW:** 23.4	**Condition:** GOOD	

SURGICAL DRUGS ADMINISTERED

	DRUGS	DOSE (mg)	ROUTE	TIME
ANESTHETIC	Propofol	8mL	IV	
	Sevoflurane	3.5%	Inhalant	
PREEMPTIVE ANALGESIC				
OTHER	Atropine Sulfate	3mL	SC	

RECOVERY NOTES: The animal recovered well without any complications. After full recovery from anesthesia, the animal (sheep) gave water + Feeds (grass). Post-operative care for 3 days with antibiotic & analgesic respectively and monitor for possible re-bleeding in the wound site.

Please ensure that all medications and procedures listed above are as stated in the approved animal used protocol.

Temperature, mucous membrane color, and depth of anesthesia should be monitored atleast every 15 minutes during anesthesia.

POST-OPERATION RECORD

DATE	TIME	SURGICAL SITE (describe, and/or use letter key below)	DRUGS GIVEN	DOSE	ROUTE	OTHER OBSERVATIONS (describe, and/or use number key below)	PAINFUL? Pain Score (Y/N)	INITIAL
6/3/2024	11:50	B	Amoxy Kel 15	2mL	I.M	1	Y	[signature]
			Ketovet	1mL	I.M			
7/3/2024	11:45	B	Amoxy Kel 15	2mL	I.M	1	Y	[signature]
			Ketovet	1mL	I.M			
8/3/2024	11:50	A	Amoxy Kel 15	2mL	I.M	1	N	[signature]
			Ketovet	1mL	I.M			

Key: qualitative assessment of surgical site and animal condition

A. Incision is clean, dry, intact
B. Incision is slightly red, clean, dry intact
C. Incision is abnormal, Please describe*

1. animal is bright, alert, responsive, and active
2. animal is quiet, alert, responsive, and less active
3. animal is lethargic and less responsive*

*contact veterinary staff

A Sheep Experimental E[illegible]taxis Model: Hemostatic Effect of Stop Nose[illegible]eds Device

Procedure Date 06/03/24 (dd/mm/yy)

Animal ID Number 01 (C03364)

1- Sheep Characteristics:

1.1) Age 24 Months

1.2) Gender ☑ M ☐ F

1.3) weight 23.4 Kg

2- Lab profile before the intervention

2.1) PT ☐☐ seconds

2.2) PTT ☐☐ seconds

3- Procedure:

3.1) Heparinized ☐ Yes ☑ No

3.2) Control wound on the right nostril (treated with manual compression that will be applied to the cartilaginous part of the nose for 15 minutes)

Time to bleeding 9:51:☐

Time to hemostasis 9:53:41

3.3) Experimental wound on the left nostril (will be treated with a Stop Nosebleeds device)

Time to bleeding 10:01:☐

Time to hemostasis 10:01:38

4-Observation after 24 hours

4.1) Re-bleeding ☐ Yes ☑ No

KING FA[illegible]AL SPECIALIST HOSPITAL AND RESEARCH C[illegible]TRE

COMPARATIVE MEDICINE DEPARTMENT

LABORATORY ANIMAL SERVICES

Surgery Record

**RAC Protocol #:** 2230013	**Animal ID#:** C03481(02)	**Microchip#:**
**Principal Investigator:** DR. Falatt	**Contact #:**	**Species:** OVINE/SHEEP ♂
**Surgeon:**	**Contact #:**	**Pre-Surgical Evaluation:**
**Surgical Procedure:** A Sheep (Ovis aries) Experimental Epistaxis Model: Hemostatic Effect of Stop Nosebleeds Device	**DATE:** [illegible]/3/24	**BW:** 22.9 **Condition:** GOOD

SURGICAL DRUGS ADMINISTERED

	DRUGS	DOSE (mg)	ROUTE	TIME
ANESTHETIC	Propofol	8mL	I.M	
	Sevoflurane	3.5%	Inhalant	
PREEMPTIVE ANALGESIC				
OTHER	Atropine Sulfate	3mL	S.C	

RECOVERY NOTES: The animal recovered well without any complications. After full recovery from anesthesia, the animal (sheep) gave water & feeds (grass). Post-operative care for 3 days with antibiotic & analgesic respectively & monitor for possible re-bleeding in the wound site.

Please ensure that all medications and procedures listed above are as stated in the approved animal used protocol.

Temperature, mucous membrane color, and depth of anesthesia should be monitored atleast every 15 minutes during anesthesia.

POST-OPERATION RECORD

DATE	TIME	SURGICAL SITE (describe, and/or use letter key below)	DRUGS GIVEN	DOSE	ROUTE	OTHER OBSERVATIONS (describe, and/or use number key below)	PAINFUL? Pain Score (Y/N)	INITIAL
6/3/2024	11:50	B	Amoxy Kel 15	2mL	I.M	1	Y	[signature]
			Ketovet	1mL	I.M			
7/3/2024	11:45	B	Amoxy Kel 15	2mL	I.M	1	Y	[signature]
			Ketovet	1mL	I.M			
8/3/2024	11:50	A	Amoxy Kel 15	2mL	I.M	1	N	[signature]
			Ketovet	1mL	I.M			

Key: qualitative assessment of surgical site and animal condition

A. Incision is clean, dry, intact
B. Incision is slightly red, clean, dry intact
C. Incision is abnormal, Please describe*

1. animal is bright, alert, responsive, and active
2. animal is quiet, alert, responsive, and less active
3. animal is lethargic and less responsive*

**contact veterinary staff*

A Sheep Experimental E[illegible]taxis Model: Hemostatic Effect of Stop Nose[illegible]eds Device

Procedure Date: 06/03/24 (dd/mm/yy)

Animal ID Number: 02 (C03841)

1- Sheep Characteristics:

1.1) Age 24 Months

1.2) Gender ☑ M ☐ F

1.3) weight 22.9 Kg

2- Lab profile before the intervention

2.1) PT ☐☐ seconds

2.2) PTT ☐☐ seconds

3- Procedure:

3.1) Heparinized ☐ Yes ☑ No

3.2) Control wound on the right nostril (treated with manual compression that will be applied to the cartilaginous part of the nose for 15 minutes)

Time to bleeding 10:41 ☐

Time to hemostasis 10:44:02

3.3) Experimental wound on the left nostril (will be treated with a Stop Nosebleeds device)

Time to bleeding 10:46 ☐

Time to hemostasis 10:46:36

4-Observation after 24 hours

4.1) Re-bleeding ☐ Yes ☑ No

KING FA[illegible]AL SPECIALIST HOSPITAL AND RESEARCH C[illegible]TRE
COMPARATIVE MEDICINE DEPARTMENT
LABORATORY ANIMAL SERVICES
Surgery Record

**RAC Protocol #:** 2230013	**Animal ID#:** CO3075(09)	**Microchip#:**
**Principal Investigator:** DR. FALAH	**Contact #:**	**Species:** OVINE/SHEEP ♂
**Surgeon:**	**Contact #:**	**Pre-Surgical Evaluation:**
**Surgical Procedure:** A Sheep (Ovis aries) Experimental Epistaxis Model: Hemostatic Effect of Stop Nosebleeds Device	**DATE:** [illegible]/3/24	**BW:** 26.4 **Condition:** GOOD

SURGICAL DRUGS ADMINISTERED

	DRUGS	DOSE (mg)	ROUTE	TIME
ANESTHETIC	Propofol	6mL	I.V	
	Sevoflurane	3.5%	Inhalant	
PREEMPTIVE ANALGESIC				
OTHER	Atropine Sulfate	3mL	S.C	

RECOVERY NOTES: The animal recovered without any complications. After full recovery from anesthesia, the anima (sheep) gave water + feeds (grass). Post-operative care for 3 days with antibiotic + analgesic respectively and monitor for possible re-bleeding in the wound site.

Please ensure that all medications and procedures listed above are as stated in the approved animal used protocol.

Temperature, mucous membrane color, and depth of anesthesia should be monitored atleast every 15 minutes during anesthesia.

POST-OPERATION RECORD

DATE	TIME	SURGICAL SITE (describe, and/or use <u>letter</u> key below)	DRUGS GIVEN	DOSE	ROUTE	OTHER OBSERVATIONS (describe, and/or use <u>number</u> key below)	PAINFUL? Pain Score (Y/N)	INITIAL
6/3/2024	11:50	B	Amoxy Kel 15	2mL	I.M	1	Y	[illegible]
			Ketovet	1mL	I.M			
7/3/2024	11:45	B	Amoxy Kel 15	2mL	I.M	1	Y	[illegible]
			Ketovet	1mL	I.M			
8/3/2024	11:50	A	Amoxy Kel 15	2mL	I.M	1	N	[illegible]
			Ketovet	1mL	I.M			

Key: qualitative assessment of surgical site and animal condition

A. Incision is clean, dry, intact
B. Incision is slightly red, clean, dry intact
C. Incision is abnormal, Please describe*

1. animal is bright, alert, responsive, and active
2. animal is quiet, alert, responsive, and less active
3. animal is lethargic and less responsive*

**contact veterinary staff*

A Sheep Experimental E[illegible]taxis Model: Hemostatic Effect of Stop Nose[illegible]eds Device

Procedure Date 06/03/24 (dd/mm/yy)

Animal ID Number 03 (C03075)

1- Sheep Characteristics:

1.1) Age 24 Months

1.2) Gender ☑ M ☐ F

1.3) weight 26.4 Kg

2- Lab profile before the intervention

2.1) PT ☐☐ seconds

2.2) PTT ☐☐ seconds

3- Procedure:

3.1) Heparinized ☐ Yes ☑ No

3.2) Control wound on the right nostril (treated with manual compression that will be applied to the cartilaginous part of the nose for 15 minutes)

Time to bleeding 11:06 ☐

Time to hemostasis 11:08:39

3.3) Experimental wound on the left nostril (will be treated with a Stop Nosebleeds device)

Time to bleeding 11:10 ☐

Time to hemostasis 11:10:43

4-Observation after 24 hours

4.1) Re-bleeding ☐ Yes ☑ No

KING FA[illegible]AL SPECIALIST HOSPITAL AND RESEARCH C[illegible]TRE
COMPARATIVE MEDICINE DEPARTMENT
LABORATORY ANIMAL SERVICES
Surgery Record

**RAC Protocol #:** 2230013	**Animal ID#:** C03068 (04)	**Microchip#:**
**Principal Investigator:** DR. FALAH	**Contact #:**	**Species:** OVINE/SHEEP
**Surgeon:**	**Contact #:**	**Pre-Surgical Evaluation:**
**Surgical Procedure:** A Sheep (Ovis aries) Experimental Epistaxis Model: Hemostatic Effect of Stop Nosebleeds Device	**DATE:** 11/3/24	**BW:** 24.0 kg **Condition:** GOOD

SURGICAL DRUGS ADMINISTERED

	DRUGS	DOSE (mg)	ROUTE	TIME
ANESTHETIC	Propofol	4mL	I.V	
	Sevoflurane	4.0%	Inhalant	
PREEMPTIVE ANALGESIC				
OTHER	Atropine sulfate	3mL	SC	

RECOVERY NOTES: The animal recovered well without any complications. After full recovery from anesthesia, the animal gave water & feeds. Post-op care with antibiotic & analgesic for 3 days will be given and monitor for possible re-bleeding in the wound site.

Please ensure that all medications and procedures listed above are as stated in the approved animal used protocol.

Temperature, mucous membrane color, and depth of anesthesia should be monitored atleast every 15 minutes during anesthesia.

POST-OPERATION RECORD

DATE	TIME	SURGICAL SITE (describe, and/or use letter key below)	DRUGS GIVEN	DOSE	ROUTE	OTHER OBSERVATIONS (describe, and/or use number key below)	PAINFUL? Pain Score (Y/N)	INITIAL
11/3/2024	10:55	B	AmoxyKel 15	2mL	I.M	1	Y	[signature]
			Voltavet	1mL	I.M			
12/3/2024	10:50	B	AmoxyKel 15	2mL	I.M	1	Y	[signature]
			Voltavet	1mL	I.M			
13/3/2024	11:10	A	AmoxyKel 15	2mL	I.M	1	N	[signature]
			Voltavet	1mL	I.M			

A. Incision is clean, dry, intact
B. Incision is slightly red, clean, dry intact
C. Incision is abnormal, Please describe*

Key: qualitative assessment of surgical site and animal condition
1. animal is bright, alert, responsive, and active
2. animal is quiet, alert, responsive, and less active
3. animal is lethargic and less responsive*

**contact veterinary staff*

A Sheep Experimental E[illegible]taxis Model: Hemostatic Effect of Stop Nose[illegible]eds Device

Procedure Date 11/03/24 (dd/mm/yy)

Animal ID Number 04 (CO3068)

1- Sheep Characteristics:

1.1) Age 24 Months

1.2) Gender ☑ M ☐ F

1.3) weight 24.0 Kg

2- Lab profile before the intervention

2.1) PT ☐☐ seconds

2.2) PTT ☐☐ seconds

3- Procedure:

3.1) Heparinized ☐ Yes ☑ No

3.2) Control wound on the right nostril (treated with manual compression that will be applied to the cartilaginous part of the nose for 15 minutes)

Time to bleeding 9:31: ☐ ✓9:31 :2:18

Time to hemostasis 9:33:18

3.3) Experimental wound on the left nostril (will be treated with a Stop Nosebleeds device)

Time to bleeding 9:25: ☐ ✓9:25 :38:6

Time to hemostasis 9:35:38

4-Observation after 24 hours

4.1) Re-bleeding ☐ Yes ☑ No

KING FA[illegible]AL SPECIALIST HOSPITAL AND RESEARCH C[illegible]NTRE
COMPARATIVE MEDICINE DEPARTMENT
LABORATORY ANIMAL SERVICES
Surgery Record

**RAC Protocol #:** 2230013	**Animal ID#:** W04038 (05)	**Microchip#:**
**Principal Investigator:** DR. Falah	**Contact #:**	**Species:** OVINE/SHEEP
**Surgeon:**	**Contact #:**	**Pre-Surgical Evaluation:**
**Surgical Procedure:** A Sheep (Ovis aries) Experimental Epistaxis Model: Hemostatic Effect of Stop Nosebleeds Device	**DATE:** 11/3/24	**BW:** 30.0kg **Condition:** GOOD

SURGICAL DRUGS ADMINISTERED

	DRUGS	DOSE (mg)	ROUTE	TIME
ANESTHETIC	Propofol	4mL	I.V	
	Sevoflurane	4.0%	Inhalant	
PREEMPTIVE ANALGESIC				
OTHER	Atropine Sulfate	3mL	SC	

RECOVERY NOTES: The animal recovered well without any complications. After full recovery from anesthesia, the animal gave water & feeds (grass). Post-op care with antibiotic & analgesic for 3 days was given and monitor for possible re-bleeding in the wound site.

Please ensure that all medications and procedures listed above are as stated in the approved animal used protocol.

Temperature, mucous membrane color, and depth of anesthesia should be monitored atleast every 15 minutes during anesthesia.

POST-OPERATION RECORD

DATE	TIME	SURGICAL SITE (describe, and/or use letter key below)	DRUGS GIVEN	DOSE	ROUTE	OTHER OBSERVATIONS (describe, and/or use number key below)	PAINFUL? Pain Score (Y/N)	INITIAL
11/3/2024	10:55	B	Amoxy Kel 15	2mL	I.M	1	Y	[signature]
			Voltavet	1mL	I.M			
12/3/2024	10:50	B	Amoxy Kel 15	2mL	I.M	1	Y	[signature]
			Voltavet	1mL	I.M			
13/3/2024	11:10	A	Amoxy Kel 15	2mL	I.M	1	N	[signature]
			Voltavet	1mL	I.M			

Key: qualitative assessment of surgical site and animal condition

A. Incision is clean, dry, intact
B. Incision is slightly red, clean, dry intact
C. Incision is abnormal, Please describe*

1. animal is bright, alert, responsive, and active
2. animal is quiet, alert, responsive, and less active
3. animal is lethargic and less responsive*

**contact veterinary staff*

A Sheep Experimental E[illegible]taxis Model: Hemostatic Effect of Stop Nose[illegible]eds Device

Procedure Date 1 1 / 0 3 / 2 4 (dd/mm/yy)

Animal ID Number 0 5 (W04038)

1- Sheep Characteristics:

1.1) Age 2 4 Months

1.2) Gender ☑ M ☐ F

1.3) weight 30 0 Kg

2- Lab profile before the intervention

2.1) PT ☐ ☐ seconds

2.2) PTT ☐ ☐ seconds

3- Procedure:

3.1) Heparinized ☐ Yes ☑ No

3.2) Control wound on the right nostril (treated with manual compression that will be applied to the cartilaginous part of the nose for 15 minutes)

✓ 9:54 :2:36

Time to bleeding 9 : 54 ☐

Time to hemostasis 9 : 56 : 36

3.3) Experimental wound on the left nostril (will be treated with a Stop Nosebleeds device)

✓ 9:58 :40.6

Time to bleeding 9 : 58 ☐

Time to hemostasis 9 : 58 : 40

4-Observation after 24 hours

4.1) Re-bleeding ☐ Yes ☑ No

KING FA[illegible]AL SPECIALIST HOSPITAL AND RESEARCH C[illegible]TRE
COMPARATIVE MEDICINE DEPARTMENT
LABORATORY ANIMAL SERVICES
Surgery Record

**RAC Protocol #:** 2230013	**Animal ID#:** 10B (06)	**Microchip#:**
**Principal Investigator:** DR. FALAH	**Contact #:**	**Species:** OVINE / SHEEP
**Surgeon:**	**Contact #:**	**Pre-Surgical Evaluation:**
**Surgical Procedure:** A Sheep (Ovis aries) Experimental Epistaxis Model: Hemostatic Effect of Stop Nosebleeds Device.	**DATE:** 11/3/24	**BW:** 31.1 Kg **Condition:** GOOD

SURGICAL DRUGS ADMINISTERED

	DRUGS	DOSE (mg)	ROUTE	TIME
ANESTHETIC	Propofol	4mL	I.V	
	Sevoflurane	4.0%	Inhalant	
PREEMPTIVE ANALGESIC				
OTHER	Atropine sulfate	3mL	SC	

RECOVERY NOTES: The animal recovered well without any complications. After full recovery from anesthesia the animal gave water & feeds. Post-op care with antibiotic & analgesic for 3 days will be given & monitor for possible re-bleeding in the wound site.

Please ensure that all medications and procedures listed above are as stated in the approved animal used protocol.

Temperature, mucous membrane color, and depth of anesthesia should be monitored atleast every 15 minutes during anesthesia.

POST-OPERATION RECORD

DATE	TIME	SURGICAL SITE (describe, and/or use letter key below)	DRUGS GIVEN	DOSE	ROUTE	OTHER OBSERVATIONS (describe, and/or use number key below)	PAINFUL? Pain Score (Y/N)	INITIAL
11/3/2024	10:55	B	Amoxy Kel 15	2mL	I.M	1	Y	[signature]
			Vottavet	1mL	I.M			
12/3/2024	10:50	B	Amoxy Kel 15	2mL	I.M	1	Y	[signature]
			Vottavet	1mL	I.M			
13/3/2024	11:10	A	Amoxy Kel 15	2mL	I.M	1	N	[signature]
			Vottavet	1mL	I.M			

Key: qualitative assessment of surgical site and animal condition

A. Incision is clean, dry, intact
B. Incision is slightly red, clean, dry intact
C. Incision is abnormal, Please describe*

1. animal is bright, alert, responsive, and active
2. animal is quiet, alert, responsive, and less active
3. animal is lethargic and less responsive*

**contact veterinary staff*

A Sheep Experimental E[illegible]taxis Model: Hemostatic Effect of Stop Nose[illegible]eds Device

Procedure Date 1 1 / 0 3 / 2 4 (dd/mm/yy)

Animal ID Number 0 6 (10B)

1- Sheep Characteristics:

1.1) Age 2 4 Months

1.2) Gender ☑ M ☐ F

1.3) weight 31 . 1 Kg

2- Lab profile before the intervention

2.1) PT ☐ ☐ seconds

2.2) PTT ☐ ☐ seconds

3- Procedure:

3.1) Heparinized ☑ Yes ☐ No

3.2) Control wound on the right nostril (treated with manual compression that will be applied to the cartilaginous part of the nose for 15 minutes)

✓ 10:17 3:10

Time to bleeding 10 : 17 : ☐

Time to hemostasis 10 : 20 : 10

3.3) Experimental wound on the left nostril (will be treated with a Stop Nosebleeds device)

✓ 10:21 :47.6

Time to bleeding 10 : 21 : ☐

Time to hemostasis 10 : 21 : 47

4-Observation after 24 hours

4.1) Re-bleeding ☐ Yes ☑ No

KING [illegible]ISAL SPECIALIST HOSPITAL AND RESEARCH [illegible]NTRE
COMPARATIVE MEDICINE DEPARTMENT
LABORATORY ANIMAL SERVICES

Surgery Record

RAC Protocol #: **2230013**	**Animal ID#:(07)** D020807	**Microchip#:**	
Principal Investigator: **DR. GORAN**	**Contact #: 45977**	**Species: OVINE/SHEEP-BARBARY**	
Surgeon: **DR. FALAH**	**Contact #: 47533**	**Pre-Surgical Evaluation:**	
Surgical Procedure: **A Sheep *(Ovis aries)* Experimental Epistaxis Model: Hemostatic Effect of Stop Nosebleeds Device.**	DATE:29/4/24	BW: 27.3 Kg	**Condition: GOOD**

SURGICAL DRUGS ADMINISTERED

	DRUGS	DOSE (mg)	ROUTE	TIME
ANESTHETIC	Propofol	8mL	I.V	
PREEMPTIVE ANALGESIC				
OTHER	Atropine Sulfate	3mL	S.C	

RECOVERY NOTES: The animal recovered well without any complications. After full recovery from anesthesia, the animal gave water and feeds. The animal will be given an antibiotic, analgesic for 3 days and pre-mix multivitamins post-operatively and monitor for any possible re-bleeding in the wound site.

Please ensure that all medications and procedures listed above are as stated in the approved animal used protocol.

Temperature, mucous membrane color, and depth of anesthesia should be monitored atleast every 15 minutes during anesthesia.

POST-OPERATION RECORD

DATE	TIME	SURGICAL SITE (describe, and/or use letter key below)	DRUGS GIVEN	DOSE	ROUTE	OTHER OBSERVATIONS (describe, and/or use number key below)	PAINFUL? Pain Score (Y/N)	INITIAL
29/4/24	10:15	B	AmoxyKel 15	2.5mL	I.M	1	Y	[illegible]
			KetoVet	1.5mL	I.M			
30/4/24	10:10	B	AmoxyKel 15	2.5mL	I.M	1	Y	[illegible]
			KetoVet	1.5mL	I.M			
01/5/24	10:10	A	AmoxyKel 15	2.5mL	I.M	1	N	[illegible]
			KetoVet	1.5mL	I.M			

Key: qualitative assessment of surgical site and animal condition

A. Incision is clean, dry, intact
B. Incision is slightly red, clean, dry intact
C. Incision is abnormal, Please describe*

1. animal is bright, alert, responsive, and active
2. animal is quiet, alert, responsive, and less active
3. animal is lethargic and less responsive*

**contact veterinary staff*

A Sheep Experimental E[illegible]taxis Model: Hemostatic Effect of Stop Nose[illegible]eds Device

Procedure Date: 2 9 / 0 4 / 2 4 (dd/mm/yy)

Animal ID Number: 0 7 (D020807)

1- Sheep Characteristics:

1.1) Age 2 4 Months

1.2) Gender ☑ M ☐ F

1.3) weight 27 . 3 Kg

2- Lab profile before the intervention

2.1) PT ☐☐ seconds

2.2) PTT ☐☐ seconds

3- Procedure:

3.1) Heparinized ☑ Yes ☐ No

3.2) Control wound on the right nostril (treated with manual compression that will be applied to the cartilaginous part of the nose for 15 minutes)

Time to bleeding 8 : 48 ☐

Time to hemostasis 8 : 51 : 32

3.3) Experimental wound on the left nostril (will be treated with a Stop Nosebleeds device)

Time to bleeding 8 : 52 ☐

Time to hemostasis 8 : 52 : 53

4-Observation after 24 hours

4.1) Re-bleeding ☐ Yes ☑ No

KING [illegible]SAL SPECIALIST HOSPITAL AND RESEARCH [illegible]NTRE
COMPARATIVE MEDICINE DEPARTMENT
LABORATORY ANIMAL SERVICES
Surgery Record

RAC Protocol #: **2230013**	**Animal ID#:(08) D02921)**	**Microchip#:**
Principal Investigator: **DR. GORAN**	**Contact #: 45977**	**Species: OVINE/SHEEP-BARBARY**
Surgeon: **DR. FALAH**	**Contact #: 47533**	**Pre-Surgical Evaluation:**
Surgical Procedure: **A Sheep *(Ovis aries)* Experimental Epistaxis Model: Hemostatic Effect of Stop Nosebleeds Device.**	**DATE:29/4/24**	**BW: 25.9 Kg** **Condition: GOOD**

SURGICAL DRUGS ADMINISTERED

	DRUGS	DOSE (mg)	ROUTE	TIME
ANESTHETIC	Propofol	8mL	I.V	
PREEMPTIVE ANALGESIC				
OTHER	Atropine Sulfate	3mL	S.C	

RECOVERY NOTES: The animal recovered well without any complications. After full recovery from anesthesia, the animal gave water and feeds. The animal will be given an antibiotic, analgesic for 3 days and pre-mix multivitamins post-operatively and monitor for any possible re-bleeding in the wound site.

Please ensure that all medications and procedures listed above are as stated in the approved animal used protocol.

Temperature, mucous membrane color, and depth of anesthesia should be monitored atleast every 15 minutes during anesthesia.

POST-OPERATION RECORD

DATE	TIME	SURGICAL SITE (describe, and/or use letter key below)	DRUGS GIVEN	DOSE	ROUTE	OTHER OBSERVATIONS (describe, and/or use number key below)	PAINFUL? Pain Score (Y/N)	INITIAL
29/4/24	10:15	B	AmoxyKel 15	2mL	I.M	1	Y	[signature]
			Ketovet	1mL	I.M			
30/4/24	10:10	B	AmoxyKel 15	2mL	I.M	1	Y	[signature]
			Ketovet	1.5mL	I.M			
01/5/24	10:10	A	AmoxyKel 15	2mL	I.M	1	N	[signature]
			Ketovet	1.5mL	I.M			

Key: qualitative assessment of surgical site and animal condition

A. incision is clean, dry, intact
B. incision is slightly red, clean, dry intact
C. incision is abnormal, Please describe*

1. animal is bright, alert, responsive, and active
2. animal is quiet, alert, responsive, and less active
3. animal is lethargic and less responsive*

**contact veterinary staff*

A Sheep Experimental E[illegible]taxis Model: Hemostatic Effect of Stop Nose[illegible]eds Device

Procedure Date 29/04/24 (dd/mm/yy)

Animal ID Number 08 (D02921)

1- Sheep Characteristics:

1.1) Age 24 Months

1.2) Gender ☑ M ☐ F

1.3) weight 25.9 Kg

2- Lab profile before the intervention

2.1) PT ☐☐ seconds

2.2) PTT ☐☐ seconds

3- Procedure:

3.1) Heparinized ☑ Yes ☐ No

3.2) Control wound on the right nostril (treated with manual compression that will be applied to the cartilaginous part of the nose for 15 minutes)

Time to bleeding 9:11:☐

Time to hemostasis 9:14:36

3.3) Experimental wound on the left nostril (will be treated with a Stop Nosebleeds device)

Time to bleeding 9:15:☐

Time to hemostasis 9:15:45

4-Observation after 24 hours

4.1) Re-bleeding ☐ Yes ☑ No

KING [illegible]SAL SPECIALIST HOSPITAL AND RESEARCH [illegible]NTRE
COMPARATIVE MEDICINE DEPARTMENT
LABORATORY ANIMAL SERVICES
Surgery Record

RAC Protocol #: **2230013**	**Animal ID#:(09) D04109**	**Microchip#:**
Principal Investigator: **DR. GORAN**	**Contact #: 45977**	**Species: OVINE/SHEEP-BARBARY**
Surgeon: **DR. FALAH**	**Contact #: 47533**	**Pre-Surgical Evaluation:**
Surgical Procedure: **A Sheep *(Ovis aries)* Experimental Epistaxis Model: Hemostatic Effect of Stop Nosebleeds Device.**	**DATE:01/5/24**	**BW: 25.4Kg** — **Condition: GOOD**

SURGICAL DRUGS ADMINISTERED

	DRUGS	DOSE (mg)	ROUTE	TIME
ANESTHETIC	Propopol	8mL	I.V	
PREEMPTIVE ANALGESIC				
OTHER	Atropine Sulfate	3mL	S.C	

RECOVERY NOTES: The animal recovered well without any complications. After full recovery from anesthesia, the animal gave water and feeds. The animal will be given an antibiotic, analgesic for 3 days and pre-mix multivitamins post-operatively and monitor for any possible re-bleeding in the wound site.

Please ensure that all medications and procedures listed above are as stated in the approved animal used protocol.

Temperature, mucous membrane color, and depth of anesthesia should be monitored atleast every 15 minutes during anesthesia.

POST-OPERATION RECORD

DATE	TIME	SURGICAL SITE (describe, and/or use letter key below)	DRUGS GIVEN	DOSE	ROUTE	OTHER OBSERVATIONS (describe, and/or use number key below)	PAINFUL? Pain Score (Y/N)	INITIAL
01/5/24	10:15	B	AmoxyKel 15	2mL	I.M	1	Y	
			Ketovet	1mL	I.M			
02/5/24	10:20	B	AmoxyKel 15	2mL	I.M	1	Y	
			Ketovet	1mL	I.M			
03/5/24	10:15	A	AmoxyKel 15	2mL	I.M	1	N	
			Ketovet	1mL	I.M			

Key: qualitative assessment of surgical site and animal condition

A. Incision is clean, dry, intact
B. Incision is slightly red, clean, dry intact
C. Incision is abnormal, Please describe*

1. animal is bright, alert, responsive, and active
2. animal is quiet, alert, responsive, and less active
3. animal is lethargic and less responsive*

****contact veterinary staff***

A Sheep Experimental E[illegible]taxis Model: Hemostatic Effect of Stop Nose[illegible]eds Device

Procedure Date 0 1 / 0 5 / 2 4 (dd/mm/yy)

Animal ID Number 0 9 (D04109)

1- Sheep Characteristics:

1.1) Age 2 4 Months

1.2) Gender ☑ M ☐ F

1.3) weight 25 . 4 Kg

2- Lab profile before the intervention

2.1) PT ☐ ☐ seconds

2.2) PTT ☐ ☐ seconds

3- Procedure:

3.1) Heparinized ☑ Yes ☐ No

3.2) Control wound on the right nostril (treated with manual compression that will be applied to the cartilaginous part of the nose for 15 minutes)

Time to bleeding 9 : 16 ☐

Time to hemostasis 9 : 22 : 40

3.3) Experimental wound on the left nostril (will be treated with a Stop Nosebleeds device)

Time to bleeding 9 : 24 ☐

Time to hemostasis 9 : 24 : 55

4-Observation after 24 hours

4.1) Re-bleeding ☐ Yes ☑ No

KING [illegible]SAL SPECIALIST HOSPITAL AND RESEARCH [illegible]ENTRE
COMPARATIVE MEDICINE DEPARTMENT
LABORATORY ANIMAL SERVICES
Surgery Record

RAC Protocol #: **2230013**	**Animal ID#:(10) Q06745**		**Microchip#:**
Principal Investigator: **DR. GORAN**	**Contact #: 45977**		**Species: OVINE/SHEEP-BARBARY**
Surgeon: **DR. FALAH**	**Contact #: 47533**		**Pre-Surgical Evaluation:**
Surgical Procedure: **A Sheep *(Ovis aries)* Experimental Epistaxis Model: Hemostatic Effect of Stop Nosebleeds Device.**	**DATE:01/5/24**	**BW: 28Kg**	**Condition: GOOD**

SURGICAL DRUGS ADMINISTERED

	DRUGS	DOSE (mg)	ROUTE	TIME
ANESTHETIC	Propofol	8mL	I.V	
PREEMPTIVE ANALGESIC				
OTHER	Atropine sulfate	3cc	S.C	

RECOVERY NOTES: The animal recovered well without any complications. After full recovery from anesthesia, the animal gave water and feeds. The animal will be given an antibiotic, analgesic for 3 days and pre-mix multivitamins post-operatively and monitor for any possible re-bleeding in the wound site.

Please ensure that all medications and procedures listed above are as stated in the approved animal used protocol.

Temperature, mucous membrane color, and depth of anesthesia should be monitored atleast every 15 minutes during anesthesia.

POST-OPERATION RECORD

DATE	TIME	SURGICAL SITE (describe, and/or use letter key below)	DRUGS GIVEN	DOSE	ROUTE	OTHER OBSERVATIONS (describe, and/or use number key below)	PAINFUL? Pain Score (Y/N)	INITIAL
01/5/24	10:15	B	AmoxyKel 15	2.5mL	I.M	1	Y	
			KetoVet	2mL	I.M			
02/5/24	10:20	B	AmoxyKel 15	2.5mL	I.M	1	Y	
			KetoVet	2mL	I.M			
03/5/24	10:15	A	AmoxyKel 15	2.5mL	I.M	1	N	
			KetoVet	2mL	I.M			

Key: qualitative assessment of surgical site and animal condition

A. incision is clean, dry, intact
B. incision is slightly red, clean, dry intact
C. incision is abnormal, Please describe*

1. animal is bright, alert, responsive, and active
2. animal is quiet, alert, responsive, and less active
3. animal is lethargic and less responsive*

****contact veterinary staff***

A Sheep Experimental [illegible]staxis Model: Hemostatic Effect of Stop Nose[illegible]eds Device

Procedure Date 01/05/24 (dd/mm/yy)

Animal ID Number 10 (Q06745)

1- Sheep Characteristics:

1.1) Age 24 Months

1.2) Gender ☑ M ☐ F

1.3) weight 28.0 Kg

2- Lab profile before the intervention

2.1) PT ☐☐ seconds

2.2) PTT ☐☐ seconds

3- Procedure:

3.1) Heparinized ☑ Yes ☐ No

3.2) Control wound on the right nostril (treated with manual compression that will be applied to the cartilaginous part of the nose for 15 minutes)

Time to bleeding 9:39:

Time to hemostasis 9:43:06

3.3) Experimental wound on the left nostril (will be treated with a Stop Nosebleeds device)

Time to bleeding 9:44:

Time to hemostasis 9:45:00

4-Observation after 24 hours

4.1) Re-bleeding ☐ Yes ☑ No
