# Supplementary material for: A goat experimental epistaxis model: Hemostatic effect of stop nosebleeds device
Source: PLoS One. 2025 Jun 10;20(6):e0324727. doi: 10.1371/journal.pone.0324727 (PMC12151359; doi:10.1371/journal.pone.0324727)
Supplement: Attachment — Submitted filename: PONE-D-24-52201_reviewer.pdf [file pone.0324727.s002.pdf]

PLOS ONE

A Goat Experimental Epistaxis Model: Hemostatic Effect of Stop Nosebleeds Device

--Manuscript Draft--

**Manuscript Number:**	PONE-D-24-52201
**Article Type:**	Research Article
**Full Title:**	A Goat Experimental Epistaxis Model: Hemostatic Effect of Stop Nosebleeds Device
**Short Title:**	Stop Nosebleeds Device
**Corresponding Author:**	Amani Abu-Shaheen, MPH<br>King Fahad Medical City<br>Riyadh, SAUDI ARABIA
**Keywords:**	Stop Nosebleeds; hemostasis; epistaxis; nasal bleeding; animal experimental
**Abstract:**	Background<br><br>To stop nasal bleeding, a Saudi medical invention called "Stop Nosebleeds" with a patent was created. It is a silicone-based, adjustable device that is applied externally to the bridge of the nose. The device could be classified as a first-aid device because it provides a temporary solution until the patient arrives at the emergency department. This study aims to examine the efficacy of the Stop Nosebleeds device on attaining hemostasis in goats with anterior nasal bleeding.<br><br>Methods<br><br>An animal experimental nasal-bleeding model was conducted on goats in collaboration between King Fahad Medical City and King Faisal Specialist Hospital and Research Center, Riyadh, Saudi Arabia. The left nostril was used as interventional wounds and the right nostril was used as control. Control wounds were treated as per routine practice with manual compression for 20 minutes. Interventional wounds were then created on the other nostril, after which it was treated with a Stop Nosebleeds device. The second group of animals was heparinized and 2 wounds were created in each animal, one of which was being treated with the Stop Nosebleeds device, while the other was being treated with manual compression.<br><br>Results<br><br>Ten adult goats were included in this study. The mean bleeding duration in the experimental group (both heparinized and non-heparinized goats) was 45.5 seconds (SD = 8.2 seconds), compared to 206.0 seconds (SD = 75.7 seconds) in the control group ($U = 0.00$, $p < 0.001$). The Stop Nose Bleeding Device stopped bleeding in 52.0 seconds and 39.0 seconds in the heparinized and non-heparinized experimental groups. While, the manual compression of epistaxis stopped bleeding in 252.5 seconds (SD = 84.7 seconds) and 159.2 seconds (SD = 15.7 seconds) in the heparinized and non-heparinized control groups, respectively.<br><br>Conclusion<br><br>The device shows promise for clinical application, potentially offering a quick and effective solution for managing nasal bleeding. Further research with larger sample sizes and comparative studies is needed to fully validate its efficacy and safety.
**Order of Authors:**	Amani Abu-Shaheen, MPH<br>Shoroq Al Janouby<br>Falah Hassan Almohanna<br>Mohsen Ayyash<br>Sumayyia Marar<br>Goran Matic

Powered by Editorial Manager® and ProduXion Manager® from Aries Systems Corporation

	Mohammed Hazazi
	Juneil Batalla
	Maaweya Awadalla
	Muaawia A. Hamza
**Additional Information:**	
**Question**	**Response**
**Financial Disclosure**<br><br>Enter a financial disclosure statement that describes the sources of funding for the work included in this submission. Review the submission guidelines for detailed requirements. View published research articles from *PLOS ONE* for specific examples.<br><br>This statement is required for submission and **will appear in the published article** if the submission is accepted. Please make sure it is accurate.<br><br>**Funded studies**<br>Enter a statement with the following details:<br>• Initials of the authors who received each award<br>• Grant numbers awarded to each author<br>• The full name of each funder<br>• URL of each funder website<br>• Did the sponsors or funders play any role in the study design, data collection and analysis, decision to publish, or preparation of the manuscript?<br><br>Did you receive funding for this work?	The author(s) received no specific funding for this work.
**Competing Interests**<br><br>Use the instructions below to enter a competing interest statement for this submission. On behalf of all authors, disclose any competing interests that could be perceived to bias this work—acknowledging all financial support and any other relevant financial or non-financial competing interests.	The authors have declared that no competing interests exist

<table>
<tr>
<td>This statement is required for submission and will appear in the published article if the submission is accepted. Please make sure it is accurate and that any funding sources listed in your Funding Information later in the submission form are also declared in your Financial Disclosure statement.

View published research articles from PLOS ONE for specific examples.

NO authors have competing interests

Enter: The authors have declared that no competing interests exist.

Authors with competing interests

Enter competing interest details beginning with this statement:

I have read the journal's policy and the authors of this manuscript have the following competing interests: [insert competing interests here]

* typeset</td>
<td></td>
</tr>
<tr>
<td>Ethics Statement

Enter an ethics statement for this submission. This statement is required if the study involved:

• Human participants
• Human specimens or tissue
• Vertebrate animals or cephalopods
• Vertebrate embryos or tissues
• Field research

Write "N/A" if the submission does not require an ethics statement.

General guidance is provided below. Consult the submission guidelines for detailed instructions. Make sure that all information entered here is included in the Methods section of the manuscript.</td>
<td>This study was approved by the Institutional Animal Care and Used Committee (IACUC) of Comparative Medicine King Faisal Specialist Hospital and Research Center, Riyadh, Saudi Arabia (RAC# 2230013). All methods were performed according to the ARRIVE (Animal Research: Reporting of In Vivo Experiments) and IACUC guidelines and regulations.</td>
</tr>
</table>

**Format for specific study types**<br><br>**Human Subject Research (involving human participants and/or tissue)**<br>• Give the name of the institutional review board or ethics committee that approved the study<br>• Include the approval number and/or a statement indicating approval of this research<br>• Indicate the form of consent obtained (written/oral) or the reason that consent was not obtained (e.g. the data were analyzed anonymously)<br><br>**Animal Research (involving vertebrate animals, embryos or tissues)**<br>• Provide the name of the Institutional Animal Care and Use Committee (IACUC) or other relevant ethics board that reviewed the study protocol, and indicate whether they approved this research or granted a formal waiver of ethical approval<br>• Include an approval number if one was obtained<br>• If the study involved *non-human primates*, add *additional details* about animal welfare and steps taken to ameliorate suffering<br>• If anesthesia, euthanasia, or any kind of animal sacrifice is part of the study, include briefly which substances and/or methods were applied<br><br>**Field Research**<br><br>Include the following details if this study involves the collection of plant, animal, or other materials from a natural setting:<br>• Field permit number<br>• Name of the institution or relevant body that granted permission	
**Data Availability**<br><br>Authors are required to make all data underlying the findings described fully available, without restriction, and from the time of publication. PLOS allows rare exceptions to address legal and ethical concerns. See the PLOS Data Policy and FAQ for detailed information.	Yes - all data are fully available without restriction

Powered by Editorial Manager® and ProduXion Manager® from Aries Systems Corporation

A Data Availability Statement describing where the data can be found is required at submission. Your answers to this question constitute the Data Availability Statement and **will be published in the article**, if accepted.<br><br>**Important:** Stating 'data available on request from the author' is not sufficient. If your data are only available upon request, select 'No' for the first question and explain your exceptional situation in the text box.<br><br>Do the authors confirm that all data underlying the findings described in their manuscript are fully available without restriction?	
**Describe where the data may be found in full sentences. If you are copying our sample text, replace any instances of XXX with the appropriate details.**<br><br>• If the data are **held or will be held in a public repository**, include URLs, accession numbers or DOIs. If this information will only be available after acceptance, indicate this by ticking the box below. For example: *All XXX files are available from the XXX database (accession number(s) XXX, XXX.*).<br>• If the data are all contained **within the manuscript and/or Supporting Information files**, enter the following: *All relevant data are within the manuscript and its Supporting Information files.*<br>• If neither of these applies but you are able to provide **details of access elsewhere**, with or without limitations, please do so. For example:<br><br>*Data cannot be shared publicly because of [XXX]. Data are available from the XXX Institutional Data Access / Ethics Committee (contact via XXX) for researchers who meet the criteria for access to confidential data.*<br><br>*The data underlying the results presented in the study are available*	The datasets used and analyzed during the current study are available from the corresponding author upon reasonable request

from (include the name of the third party and contact information or URL).<br>• This text is appropriate if the data are owned by a third party and authors do not have permission to share the data.<br><br>* typeset	
Additional data availability information:	

Manuscript Click here to access/download;Manuscript;@Goat study.docx

A Goat Experimental Epistaxis Model: Hemostatic Effect of Stop Nosebleeds Device

Authors:

Amani Abu-Shaheen* [1], Shoroq Al Janouby [2], Falah Hassan Almohanna [3], Mohsen Ayyash [4], Sumayyia Marar [1], Goran Matic [3], Mohammed Hazazi [3], Juneil Batalla [3], Maaweya Awadalla [1], Muaawia A. Hamza 1

Affiliations:

1. Research Center, King Fahad Medical City, Riyadh Second Health Cluster, Riyadh, Saudi Arabia
2. Not affiliated
3. Department of Comparative Medicine, King Faisal Specialist Hospital and Research Centre, Riyadh, Saudi Arabia
4. School of Mathematical Sciences, University Sains Malaysia, 11800 USM, Pinang, Malaysia

Conflict of Interest:

The authors declare no conflicts of interest.

Source of Funding:

Grant management department, Research Center, King Fahad Medical City, Riyadh, Saudi Arabia.

Acknowledgments

We are grateful to the King Fahad Medical City Research Center, Riyadh, Saudi Arabia, for providing us with the financial support to complete this project (IRF# 022-036).

Data availability

The datasets used and analyzed during the current study are available from the corresponding author upon reasonable request

Corresponding Author:

***Amani Abu-Shaheen**

Chairperson, Department of Scientific Writing and Biostatistics

Research Center

King Fahad Medical City

Riyadh Second Health Cluster

P.O. box. 59046, Riyadh 11525

Kingdom of Saudi Arabia

aabushaheen@kfmc.med.sa

(+966) 11 288 9999 Ext: 10851

ORCID ID: https://orcid.org/0000-0002-1019-5548

Abstract

Background: To stop nasal bleeding, a Saudi medical invention called "Stop Nosebleeds" with a patent was created. It is a silicone-based, adjustable device that is applied externally to the bridge of the nose. The device could be classified as a first-aid device because it provides a temporary solution until the patient arrives at the emergency department. This study aims to examine the efficacy of the Stop Nosebleeds device on attaining hemostasis in goats with anterior nasal bleeding. **Methods:** An animal experimental nasal-bleeding model was conducted on goats in collaboration between King Fahad Medical City and King Faisal Specialist Hospital and Research Center, Riyadh, Saudi Arabia. The left nostril was used as interventional wounds and the right nostril was used as control. Control wounds were treated as per routine practice with manual compression for 20 minutes. Interventional wounds were then created on the other nostril, after which it was treated with a Stop Nosebleeds device. The second group of animals was heparinized and 2 wounds were created in each animal, one of which was being treated with the Stop Nosebleeds device, while the other was being treated with manual compression. **Results:** Ten adult goats were included in this study. The mean bleeding duration in the experimental group (both heparinized and non-heparinized goats) was 45.5 seconds (SD = 8.2 seconds), compared to 206.0 seconds (SD = 75.7 seconds) in the control group ($U = 0.00$, $p < 0.001$). The Stop Nose Bleeding Device stopped bleeding in 52.0 seconds and 39.0 seconds in the heparinized and non-heparinized experimental groups. While, the manual compression of epistaxis stopped bleeding in 252.5 seconds (SD = 84.7 seconds) and 159.2 seconds (SD = 15.7 seconds) in the heparinized and non-heparinized control groups, respectively. **Conclusion:** The device shows promise for clinical application, potentially offering a quick and effective solution for managing nasal bleeding. Further research with larger sample sizes and comparative studies is needed to fully validate its efficacy and safety.

Introduction

Epistaxis is active bleeding from the nasal mucosa (1,2). The major bleeding location determines whether the epistaxis is anterior or posterior (3). The anterior nasal septum causes up to 95% epistaxis, and the majority of epistaxis occurs in Kisselbach's plexus, which is positioned over the anterior-inferior section of the nasal septum (4,5).

Epistaxis is one of the most prevalent otolaryngology emergencies, and it can be life-threatening. Every year, an estimated 5% to 10% of the world's population suffers from epistaxis (1,2). Up to 60% of the population has experienced spontaneous epistaxis, with the highest rates in those under the age of 10 and those over the age of 50 (2).

Epistaxis can be caused by a variety of local or systemic factors. Trauma, upper airway infections, inhaling cold and dry air, nasal allergies, foreign body insertion, septal perforation or deviation, neoplasia, and chemical irritants are all common local causes of epistaxis. Hypertension, coagulopathies, especially those caused by anticoagulant or antiplatelet adhesion medicine, renal failure, alcoholism, and vascular abnormalities are all common systemic causes (3,4).

Pressing the nostrils, putting a cold compress on the bridge of the nose, hot water irrigation, anterior nasal packing, cauterization, hemostatic or vasoconstriction medicines, and cryotherapy are all useful treatments for epistaxis (1,3,4). These approaches, however, can increase the risk of infection, septal perforation, and aspiration. As a result, researchers are continuing to search for new ways to manage epistaxis (6-8).

The 2020 ‘‘Clinical Practice Guideline: Nosebleed (Epistaxis)’’ was produced to guide healthcare practitioners on aspects related to nosebleeds. The guidelines include recommendations for first-line management to treat active bleeding in patients. This recommendation was compression of the lower

third of the nose for 5 minutes or longer, which proved to be very effective (10). We developed a Saudi medical invention “Stop Nosebleeds” device with a patent; to stop epistaxis. Stop Nosebleeds does not interfere with the components of the traditional coagulation route. As a result, it could be useful in people who have normal hemostatic parameters as well as those who have primary or secondary hemostatic disorders. Moreover, the device might be considered a first-aid tool because it provides a temporary remedy until the patient arrives at the emergency room. The evaluation of the different epistaxis management models has been discussed in many studies (11-19). However, the effectiveness of different management models and the clinical efficacy of different treatments still need further investigation (20,21). Thus, this study aims to examine the efficacy of the Stop Nosebleeds device on attaining hemostasis in goats with anterior nasal bleeding.

Materials and methods

An animal experimental nasal-bleeding model was conducted using the Stop Nosebleeds device (Figure 1) in collaboration between the Research Center, King Fahad Medical City, Riyadh, Saudi Arabia, and the Laboratory Animal Service, Department of Comparative Medicine King Faisal Specialist Hospital and Research Center, Riyadh, Saudi Arabia.

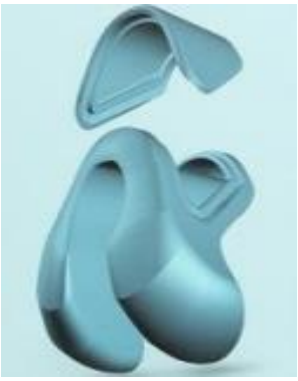

Figure 1. Stop Nosebleeds device used to stop epistaxis.

The device is an external silicon-based, adjustable device for various nose forms that is applied externally over the nasal bridge (Figure 2). The device increases the speed of blood clotting in the nose area by lowering the temperature of the exterior walls, which is induced by a chemical reaction inside the device's cavity. The device works by rubbing the ends of the piece together by hand, causing the water bag in the device's chamber to blast and the ammonium nitrate crystals to start a chemical reaction, which leads to a rapid drop in the temperature. By this unique mechanism of action, the outer walls of the device reach four degrees Celsius and inflate within 45 seconds, which speeds up blood clotting due to the decrease in the temperature and the compressing effect of the device on the nose.

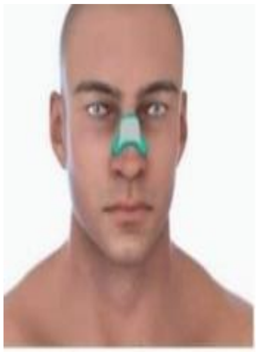

Figure 2. Stop the Nosebleeds device by applying pressure and cooling over the nasal bridge.

Ten (10) male local goats were used in this study. Domestic goats were chosen as the experimental animals because all animals and their nasal blood flow most closely resemble that of human beings. The goat’s age was between 6 and 12 months. Goats with any disease or abnormalities were excluded from this study. The animals were purchased locally and kept in a room at a constant temperature (22°C ± 4°C) with a 12-hour light/dark cycle, 55 ± 5 % humidity, normal diet, and free access to tap water several days before the investigation and were fasted overnight before any procedures. The study protocol, housing, and care for animals followed the animal care and use committee at the Department of Comparative Medicine King Faisal Specialized Hospital and Research Center, Riyadh, Saudi Arabia.

Study protocol

The study was conducted at the Laboratory Animal Service, Department of Comparative Medicine King Faisal Specialist Hospital and Research Center, Riyadh, Saudi Arabia. Ten goats were recruited in the current study, such that the left nostril was used as the experimental group, and the right nostril was used as the control (Figure 3). The animals used in this study were indigenous Saudi goat breeds locally known as Harri (species: Capra hircus).

Five animals were generally anesthetized using sevoflurane. Standardized, full-thickness, mucosal wounds were created with a 3-mm surgical punch on the nasal anterior septum of the goats. The surgical punch was applied to the nasal mucosa over the right and left anterior part of the nasal septum using slight pressure. The punch was rotated 90° clockwise and 90° counterclockwise to yield a full-thickness mucosal cut without harming the septal cartilage. This resulted in bleeding within 10 to 20 seconds of injury in all cases. Only the surgical punches resulted in consistent and uniform wounds that resulted in bleeding for at least 30 seconds in the unheparinized animal. Control wounds were treated as per routine practice with manual compression that was applied to the cartilaginous part of the nose for 20 minutes. The time to hemostasis was determined for this control wound. A wound was then created on the other nostril and allowed to bleed for 30 seconds, after which it was treated with a Stop Nosebleeds device. Five new goats were heparinized (with an intravenous bolus of heparin sulfate 100 international units/kg) and went through the same anesthesia protocol. Two wounds were then created in each animal, one of which was treated with the Stop Nosebleeds device, while the other was treated with manual compression. After completing the experiment, the animals were allowed to recover from anesthesia and then transferred to the postoperative care room. Vital signs (temperature, heart rate, respiratory rate, mucous membrane (gums) color and capillary refill time) and animal’s activities/behaviors were assessed and documented on the post-operative recovery room by

the veterinary technician. Nasal wounds were observed for 24 hours for any sign of re-bleeding. After study's completion, the animals were return to the comparative medicine department.

The data were collected using a predesigned data collection sheet. The data collection sheet encompasses 6 sections: goat characteristics including the age and gender of the goats, lab profile before the intervention, procedures without heparin and with heparin for both intervention and control wounds, and observation of re-bleeding after 24 hours.

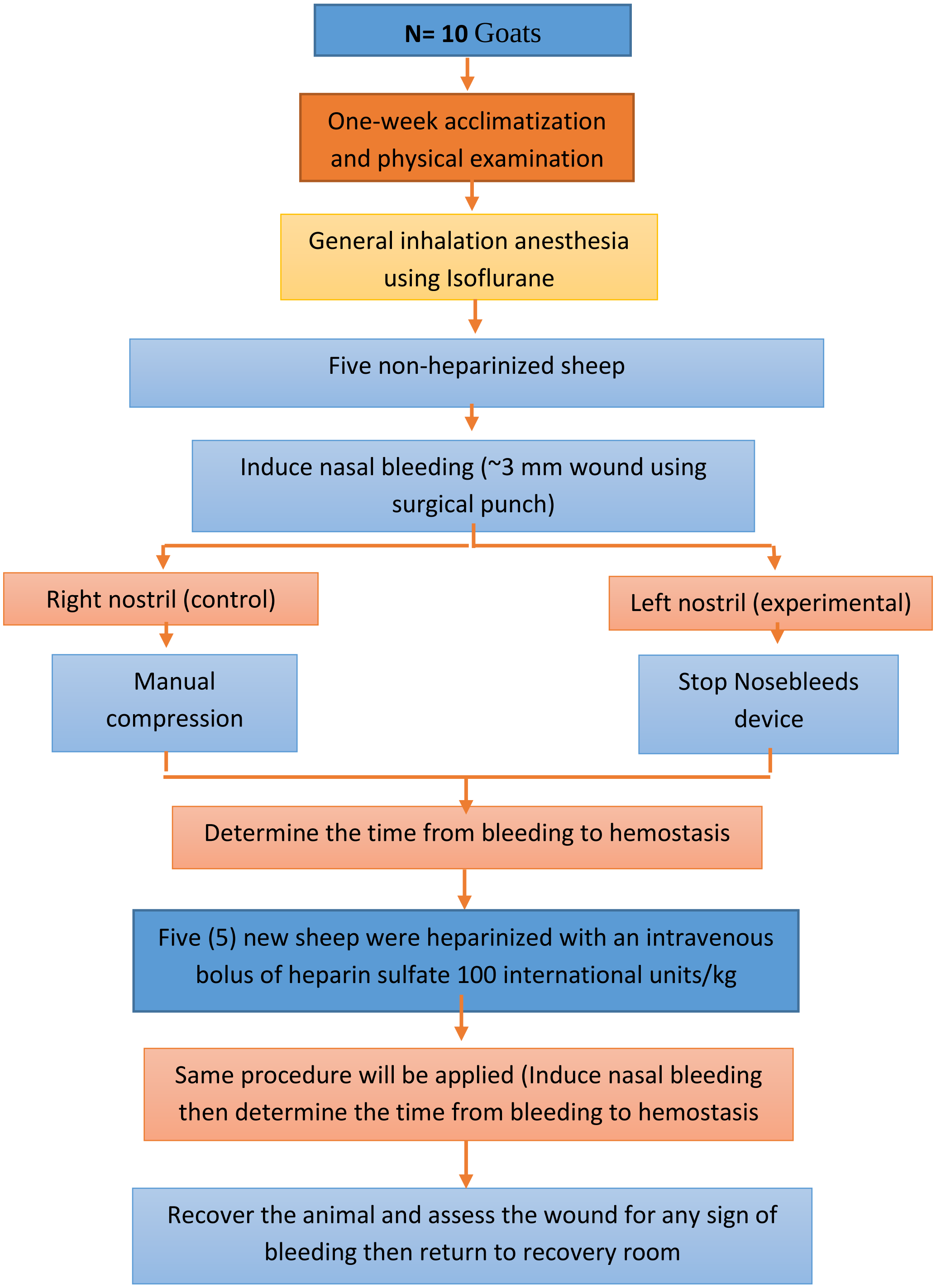
N= 10 Goats
One-week acclimatization and physical examination
General inhalation anesthesia using Isoflurane
Five non-heparinized sheep
Induce nasal bleeding (~3 mm wound using surgical punch)
Right nostril (control)
Left nostril (experimental)
Manual compression
Stop Nosebleeds device
Determine the time from bleeding to hemostasis
Five (5) new sheep were heparinized with an intravenous bolus of heparin sulfate 100 international units/kg
Same procedure will be applied (Induce nasal bleeding then determine the time from bleeding to hemostasis
Recover the animal and assess the wound for any sign of bleeding then return to recovery room

Figure 3. Roadmap of the study protocol and procedures.

Outcomes

The primary outcome was the proportion of wounds in which complete hemostasis is achieved. The secondary outcome was the time to complete hemostasis in both intervention and control wounds (in seconds). Hemostasis was measured from the moment of injury and was considered complete when no visual blood pooling was noted.

Ethical consideration

This study was approved by the Institutional Animal Care and Used Committee (IACUC) of Comparative Medicine King Faisal Specialist Hospital and Research Center, Riyadh, Saudi Arabia (RAC# 2230013). All methods were performed according to the ARRIVE (Animal Research: Reporting of In Vivo Experiments) and IACUC guidelines and regulations.

Sample size estimate

We conducted a power analysis to determine the required sample size (22,23). Assuming a standard deviation of 1.25 minutes in bleeding time for each goat and aiming to detect a 2-minute mean difference between the experimental and control groups, the effect size (D) was calculated to be 1.6 (D = difference in means / standard deviation). Therefore, with a significance level of 5%, a power of 90%, a two-sided test, and an effect size of 1.6, the required sample size is 10 goats (22).

Statistical Analysis

Study variables were described using means and standard deviations (SDs). To compare bleeding duration or bleeding time between the experimental and control groups, as well as between heparinized

and non-heparinized goats, the Mann-Whitney U test was used. Statistical significance was defined at p-values less than 0.05. Data analyses were conducted using the Statistical Package for the Social Sciences (SPSS) version 26.0.

Results

Ten adult goats, aged 6-12 months with an average weight of 26.44 kg (SD = 2.72 kg), were included in this study. Table 1 presents the bleeding times measured for wounds on the right (control) and left (experimental) nostrils. The mean bleeding duration in the experimental group (both heparinized and non-heparinized goats) was 45.5 seconds (SD = 8.2 seconds), compared to 206.0 seconds (SD = 75.7 seconds) in the control group. The results of the Mann-Whitney test indicate that the mean bleeding time in wounds treated with the Stop Bleeding Device (intervention) was significantly shorter than in the control group ($U = 0.00$, $p < 0.001$). During the 24-hour observation period, none of the wounds experienced recurrent bleeding.

Table 1. Bleeding times in goats with epistaxis on the right and left nostril

**Goat ID**	**Left Nostril (Experimental Group)**	**Right Nostril (Control Group)**
1	38	161
2	36	182
3	43	159
4	38	138
5	40	156
6	47	190
7	53	212
8	45	216
9	55	400
10	60	246
Mean (SD)	45.5 (8.2)	206.0 (75.7)
Median (Range)	44.0 (24)	186.0 (262)
Mann-Whitney Test Statistic (U)	0.00	
P-value	< 0.001	

Time is presented in seconds		

The Stop Nose Bleeding Device stopped bleeding in 52.0 seconds (SD = 5.4 seconds) and 39.0 seconds (SD = 2.6 seconds) in the heparinized and non-heparinized experimental groups, respectively. The difference in average bleeding times between the heparinized and non-heparinized interventional groups was statistically significant (p = 0.004). The manual compression of epistaxis stopped bleeding in 252.5 seconds (SD = 84.7 seconds) and 159.2 seconds (SD = 15.7 seconds) in the heparinized and non-heparinized control groups, respectively, with the difference also being statistically significant (p = 0.004). The Stop Nose Bleeding Device was effective in reducing bleeding duration compared to manual compression, with this effect being more pronounced in non-heparinized goats (p = 0.004) (Table 2).

Table 2. Mean bleeding duration in heparinized and no heparinized goats

	Left Nostril	Right Nostril
Non-Heparinized (n = 5)	39.0 (2.6)	159.2 (15.7)
Heparinized (n = 5)	52.0 (5.4)	252.8 (84.7)
P-value (Mann-Whitney Test)	0.004	0.004

As for postoperative care, all goats received three doses of antibiotic (Amoxykel, 15 mg/kg via intramuscular route) and analgesic (Ketovet, 50 mg/kg via intramuscular route) over three days (one dose per day) and were monitored for potential wound re-bleeding.

Discussion

Epistaxis is a frequent and sometimes severe medical condition. Various hemostatic agents and devices have been studied to manage epistaxis effectively, particularly in animal models, to understand their efficacy and safety before human application (1,5). This study on goats suggests that the Stop Nosebleeds Device has a significant hemostatic effect on epistaxis. The device was found to be effective in shortening the duration of bleeding compared to manual compression in a goat epistaxis model.

The findings of this study align with previous studies that have demonstrated the efficacy of various hemostatic devices and agents in reducing bleeding times in animal models (1,5,24,25). For instance, Kurtaran et al. (1) indicated that the use of Ankaferd BloodStopper (ABS) as a topical hemostatic agent significantly reduced bleeding duration compared to the isotonic saline solution in a rabbit epistaxis model. Similarly, Yurttaş et al. (24) showed that ABS and microporous polysaccharide hemospheres (MPH) are both safe, effective, and quick-acting hemostatic agents for treating epistaxis, with MPH showing greater efficacy in reducing hemostasis time and nasal bleeding. Kelleş et al. (25) suggested that the topical nasal application of ABS controls epistaxis more quickly than gelatin foam and adrenaline + lidocaine in a rabbit bleeding model, potentially leading to better patient outcomes.

Our findings are consistent with those of Singer et al. (5), who demonstrated that oxidized cellulose achieves effective hemostasis in a standardized porcine epistaxis model, reducing time to hemostasis

with fewer applications. Collectively, these studies indicate that various hemostatic agents, including tacrolimus, oxidized cellulose, ABS, MPH, and OCA, show promise in effectively managing epistaxis. These agents generally outperform traditional methods like nasal packing, providing faster and more reliable hemostasis. Therefore, the current study provides additional evidence of the effectiveness of the Stop Nosebleeds Device in reducing the duration of bleeding in a goat model compared to traditional manual compression procedures.

The administration of antibiotics and analgesics postoperatively ensured that no infections or significant pain were experienced by the animals, which could have influenced the outcomes. The use of Amoxykel and Ketovet was consistent with veterinary guidelines for postoperative care (10, 29). No re-bleeding incidents were observed during the 24-hour monitoring period, indicating that the hemostasis achieved was stable and effective, consistent with other animal epistaxis models (1, 3).

The marked decrease in bleeding time suggests that the Stop Nosebleeds Device may be highly effective for managing epistaxis in clinical settings. Achieving rapid hemostasis is crucial in both emergency and routine care to prevent complications from prolonged bleeding. This device could minimize the necessity for more invasive procedures and potentially enhance patient outcomes. However, this study is subject to some limitations, including the small sample size and the lack of long-term follow-up to study the effects of the device and ensure sustained hemostasis and safety.

While the findings are robust, future studies on humans are needed to confirm the current results. Moreover, it would be beneficial to investigate the long-term effects of the Stop Nosebleeds Device to ensure sustained hemostasis and safety. Future research should explore the comparative effectiveness of the Stop Nosebleeds Device against other established hemostatic agents and devices. This could include head-to-head trials in various clinical and animal models to better understand its relative

advantages. Investigating the mechanisms by which the device achieves hemostasis could also provide valuable insights and potentially lead to further improvements.

Our findings are expected to serve as the foundation for future randomized controlled trials on humans, shedding light on the need for those patients to have a friendly use device as a first-aid tool to provide a temporary solution until the patient arrives at the emergency room.

Conclusion

This study suggests that the Stop Nosebleeds Device significantly reduces bleeding time in a goat model of epistaxis, demonstrating its potential as an effective hemostatic intervention. These results are supported by similar findings in previous studies involving other hemostatic devices and animal models. The device shows promise for clinical application, potentially offering a quick and effective solution for managing nasal bleeding. Further research with larger sample sizes and comparative studies is needed to fully validate its efficacy and safety.

References

1. Kurtaran H, Ark N, Ugur SK, et al. Effects of a topical hemostatic agent on an epistaxis model in rabbits. Curr Ther Res. 2010; 71:105-110.
2. Robertson S, Kubba H. Long-term effectiveness of antiseptic cream for recurrent epistaxis in childhood: five-year follow up of a randomized, controlled trial. J Laryngol Otol. 2008; 122:1084-1087.
3. Teker AM, Korkut AY, Kahya V, Gedikli O. Prospective, randomized, controlled clinical trial of Ankaferd Blood Stopper in patients with acute anterior epistaxis. Eur Arch Otorhinolaryngol. 2010; 267:1377-1381.
4. Gifford TO, Orlandi RR. Epistaxis. Otolaryngol Clin North Am. 2008; 41:525-536.
5. Singer AJ, McClain SA, Katz A. A porcine epistaxis model: hemostatic effects of octyl cyanoacrylate. Otolaryngol Head Neck Surg. 2004; 130:553-557.
6. Vaghela. (2005). Using a swimmer's nose clip in the treatment of epistaxis in the A&E department. Accident and Emergency Nursing, 13(4), 261–263. https://doi.org/10.1016/j.aaen.2005.07.006
7. Hajimaghsoudi M, Largani HA, Baradaranfar MH, Aghabagheri M, Jafari MA, Saeedi M. A novel method for epistaxis management: Randomized clinical trial comparing nose clip with manual compression. Am J Emerg Med. 2018 Jan;36(1):149-150. doi: 10.1016/j.ajem.2017.07.025. Epub 2017 Jul 6. PMID: 28736091.
8. Abdul-Hamid A, Qureishi A, Martinez-Devesa P, Almeyda R. Giving epistaxis two fingers. Clin Otolaryngol. 2019; 44:882–883. https://doi.org/10.1111/coa.13268

9. Womack, Kropa, J., & Jimenez Stabile, M. (2018). Epistaxis: Outpatient Management. American Family Physician, 98(4), 240–245.
10. Tunkel, Anne, S., Payne, S. C., Ishman, S. L., Rosenfeld, R. M., Abramson, P. J., Alikhaani, J. D., Benoit, M. M., Bercovitz, R. S., Brown, M. D., Chernobilsky, B., Feldstein, D. A., Hackell, J. M., Holbrook, E. H., Holdsworth, S. M., Lin, K. W., Lind, M. M., Poetker, D. M., Riley, C. A., … Monjur, T. M. (2020). Clinical Practice Guideline: Nosebleed (Epistaxis) Executive Summary. Otolaryngology-Head and Neck Surgery, 162(1), 8–25. https://doi.org/10.1177/0194599819889955
11. Reuben et al. The Use of Tranexamic Acid to Reduce the Need for Nasal Packing in Epistaxis (NoPAC): Randomized Controlled Trial Annals of Emergency Medicine. Volume 77, no. 6: June 2021
12. Joseph J, Martinez-Devesa P, Bellorini J, et al. Tranexamic acid for patients with nasal haemorrhage (epistaxis). Cochrane Database Syst Rev. 2018:CD004328
13. Zahed et al. A new and rapid method for epistaxis treatment using injectable form of tranexamic acid topically: a randomized controlled trial. Am J Emerg Med. 2013 Sep;31(9):1389-92. doi: 10.1016/j.ajem.2013.06.043. Epub 2013 Jul 30.
14. Murray S, Mendez A, Hopkins A, El-Hakim H, Jeffery CC, Côté DWJ. Management of Persistent Epistaxis Using Floseal Hemostatic Matrix vs. traditional nasal packing: a prospective randomized control trial. J Otolaryngol Head Neck Surg. 2018 Jan 8;47(1):3. doi: 10.1186/s40463-017-0248-5. PMID: 29310703; PMCID: PMC5759868.
15. Shargorodsky J, Bleier BS, Holbrook EH, et al. Outcomes Analysis in Epistaxis Management: Development of a Therapeutic Algorithm. Otolaryngology–Head and Neck Surgery. 2013;149(3):390-398. doi:10.1177/0194599813492949

16. Fukutsuji K, et al. Superselective angiographic embolization for intractable epistaxis. Acta Otolaryngol 2008;128(5):556–60.
17. Viehweg TL, Roberson JB, Hudson JW. Epistaxis: diagnosis and treatment. J Oral Maxillofac Surg 2006;64(3):511–8.
18. Zahed R, Moharamzadeh P, AlizadehArasi S, Ghasemi A, Saeedi M. A new and rapid method for epistaxis treatment using injectable form of tranexamic acid topically: a randomized controlled trial. Am J Emerg Med 2013 Sep 30;31(9):1389–92
19. Tanner R, Harney MS. The initial management of epistaxis. Ir Med J. 2015 Apr;108(4):123-4. PMID: 26016308.
20. Newton et al. An outcomes analysis of anterior epistaxis management in the emergency department. Journal of Otolaryngology-Head and Neck Surgery (2016) 45:24
21. Koçak, Bilece, Z. T., Keskin, M., Ulusoy, H. A., Koç, A. K., & Kaya, K. H. (2021). Comparison of topical treatment methods used in recurrent anterior epistaxis: a randomized clinical trial. Brazilian Journal of Otorhinolaryngology, 87(2), 132–136. https://doi.org/10.1016/j.bjorl.2019.07.002
22. Festing MF, Altman DG. Guidelines for the design and statistical analysis of experiments using laboratory animals. ILAR journal. 2002 Jan 1;43(4):244-58.
23. Charan J, Kantharia N. How to calculate sample size in animal studies? Journal of Pharmacology and Pharmacotherapeutics. 2013 Dec;4(4):303-6.
24. Yurttas V, Sereflican M, Terzi EH, Ozyalvaçlı G, Kazaz H. Comparison of microporous polysaccharide hemospheres and Ankaferd Blood Stopper in a rabbit epistaxis model. European Archives of Oto-Rhino-Laryngology. 2016 Apr;273:933-8. https://doi.org/10.1007/s00405-015-3692-8

25. Kelleş M, Kalcioglu MT, Samdanci E, Selimoglu E, Iraz M, Miman MC, Haznedaroglu IC. Ankaferd blood stopper is more effective than adrenaline plus lidocaine and gelatin foam in the treatment of epistaxis in rabbits. Current therapeutic research. 2011 Oct 1;72(5):185-94. https://doi.org/10.1016/j.curtheres.2011.08.002
26. Dupuis-Girod S, Fargeton AE, Grobost V, Rivière S, Beaudoin M, Decullier E, Bernard L, Bréant V, Colombet B, Philouze P, Bailly S. Efficacy and safety of a 0.1% tacrolimus nasal ointment as a treatment for epistaxis in hereditary hemorrhagic telangiectasia: a double-blind, randomized, placebo-controlled, multicenter trial. Journal of Clinical Medicine. 2020 Apr 26;9(5):1262. https://doi.org/10.3390/jcm9051262
27. Khurmi AH, Alsamih MF, Alhejji KA, Al Hamoud FA, Obaidi EA, Salat FM, Al Jama DA, Tashkandi RE, Algannas TS, Alanazi AS. Acute epistaxis management by hemostatic agents a systematic review. International Journal of Medicine in Developing Countries. 2024 Jan 4;8(1):460-. https://doi.org/10.24911/ijmdc.51-1701700555
28. Te Grotenhuis R, van Grunsven PM, Heutz WM, Tan EC. Use of hemostatic nasal plugs in emergency medical services in the Netherlands: a prospective study of 33 cases. Prehospital Emergency Care. 2018 Jan 2;22(1):91-8. https://doi.org/10.1080/10903127.2017.1335816
29. Raber JM, Niekrasz M, Linkenhoker J, Perdue KA. Veterinary Care. Management of Animal Care and Use Programs in Research, Education, and Testing. 2017 Sep 7:733-70. https://www.ncbi.nlm.nih.gov/books/NBK54052/
