# Supplementary material for: A goat experimental epistaxis model: Hemostatic effect of stop nosebleeds device
Source: PLoS One. 2025 Jun 10;20(6):e0324727. doi: 10.1371/journal.pone.0324727 (PMC12151359; doi:10.1371/journal.pone.0324727)
Supplement: Attachment — Submitted filename: PONE-D-24-52201-rev D.docx [file pone.0324727.s003.docx]

The manuscript titled "A Goat Experimental Epistaxis Model: Hemostatic Effect of Stop Nosebleeds Device" addresses a clinically relevant issue by testing a novel hemostatic device. The study design and results are promising; however, there are areas where the manuscript could be improved.

**Abstract:**

The abstract effectively summarizes the study but would benefit from specifying areas requiring further research, such as long-term outcomes or human clinical trials. Additionally, it should explicitly acknowledge the inherent limitation of translating findings from a goat model to human clinical applications to provide a more balanced perspective.

**Introduction:**

1. While the introduction highlights the importance of managing epistaxis and introduces the device, it lacks a robust justification for selecting goats as the model organism. Including a comparison to other animal models in the literature would strengthen this section.
2. The novelty of the "Stop Nosebleeds" device is mentioned, but it would be helpful to include a brief comparison with existing hemostatic devices or agents.

**Material and methods:**

1. Device Mechanism: The cooling mechanism's contribution to hemostasis is not directly validated. Adding data on nasal tissue temperature or citing similar studies could substantiate this claim.
2. Bleeding Time Measurement: The criteria for determining complete hemostasis (absence of visible blood pooling) should be supported with additional references or validation.

**Results:**

1. The variability in bleeding times (e.g., control group SD = 75.7 seconds) raises questions about potential inconsistencies in wound creation or animal responses. Addressing this variability in the discussion would be useful.
2. The results rely solely on text and tables, which, while clear, lack complementary visual aids like graphs or charts.

**Discussion:**

1. Indirect comparisons: The discussion summarizes the findings of other hemostatic agents but does not directly compare their outcomes (e.g., bleeding times, efficacy rates) to the Stop Nosebleeds device. It lacks quantitative comparisons, such as how the bleeding times achieved with ABS or MPH differ from those achieved with the Stop Nosebleeds device.
2. No focus on device uniqueness: While it mentions similarities to other devices, the unique advantages or disadvantages of the Stop Nosebleeds device (e.g., simplicity, cooling mechanism) compared to ABS, MPH, or oxidized cellulose are not highlighted.
3. Contextualize for clinical use: Address why the Stop Nosebleeds device could be advantageous in real-world scenarios (e.g., first-aid use, portability, or safety in anticoagulated patients).
