# Supplementary material for: A goat experimental epistaxis model: Hemostatic effect of stop nosebleeds device
Source: PLoS One. 2025 Jun 10;20(6):e0324727. doi: 10.1371/journal.pone.0324727 (PMC12151359; doi:10.1371/journal.pone.0324727)
Supplement: Attachment — Submitted filename: Review of the document.docx [file pone.0324727.s004.docx]

**Review of the document: A Goat Experimental Epistaxis Model: Hemostatic Effect of Stop Nosebleeds Device**

The topic is quite interesting but the document is not in the format of the journal nor does it meet the minimum parameters of the journal. Be careful with the line number. The abstract exceeds 300 words. There is no homogeneity throughout the document. It is very disorderly. The title of materials and methods is missing, as well as the guide for the protocol. The figures should be put “Fig.1”, “Fig.2” etc. The tables are not in the correct format. There is a lack of coherence in the writing. Only 20.6% are citations from 2019 to 2024 which means that 79.3% are sources under 5 years old. Citations number 26, 27 and 28 are not listed in the bibliography. All citations are not found with Vancouver standards, which is what the journal indicates, but a mixture of this with apa standards. Be careful, and also take into account the homogeneity in these same.
