# Supplementary material for: A goat experimental epistaxis model: Hemostatic effect of stop nosebleeds device
Source: PLoS One. 2025 Jun 10;20(6):e0324727. doi: 10.1371/journal.pone.0324727 (PMC12151359; doi:10.1371/journal.pone.0324727)
Supplement: Attachment — Submitted filename: Response to Reviewers.docx [file pone.0324727.s006.docx]

18 March, 2025

The Editor

PLOS ONE

**Subject:** Submission of the first revision of Manuscript: PONE-D-24-52201
Dear Editor, 

Thank you very much for the evaluation and consideration of our manuscript titled “A Goat Experimental Epistaxis Model: Hemostatic Effect of Stop Nosebleeds Device”, submitted for exclusive consideration for publication with PLOS One.

We greatly appreciate the insightful comments and feedback provided by the external referees. We have made revisions throughout the manuscript in response to the comments and included an itemized response below. All changes in the manuscript have been noted in track changes.

We believe that the modifications have strengthened the manuscript and successfully prepared for publication.  

We appreciate the time and effort spent in this peer review process and thank you for your continued consideration of our work.  

Please contact us if you need any further revisions or information.

Authors

**Response to Reviewer Comments**

**Reviewer #1:**

The idea is fair new. Please check the document with track changes.

**Response:** Thank you for the comments. All suggested edits have been incorporated in the revised manuscript.

**Reviewer #2:**

The manuscript titled "A Goat Experimental Epistaxis Model: Hemostatic Effect of Stop Nosebleeds Device" addresses a clinically relevant issue by testing a novel hemostatic device. The study design and results are promising; however, there are areas where the manuscript could be improved.
Abstract:
The abstract effectively summarizes the study but would benefit from specifying areas requiring further research, such as long-term outcomes or human clinical trials. Additionally, it should explicitly acknowledge the inherent limitation of translating findings from a goat model to human clinical applications to provide a more balanced perspective.
**Response:** We appreciate the valuable feedback. The suggested points have been included in the abstract of the revised manuscript.
Introduction:
1.While the introduction highlights the importance of managing epistaxis and introduces the device, it lacks a robust justification for selecting goats as the model organism. Including a comparison to other animal models in the literature would strengthen this section.

**Response:** Thank you for your suggestion. We have added a few lines in the introduction that justify the selection of goats in this study in comparison to other animal models.

2.The novelty of the "Stop Nosebleeds" device is mentioned, but it would be helpful to include a brief comparison with existing hemostatic devices or agents.
**Response:** Thank you for the comment. In the revised manuscript we have included a brief comparison of Stop Nosebleeds device with traditional hemostatic devices such as anterior nasal packaging.

Material and methods:

1.Device Mechanism: The cooling mechanism's contribution to hemostasis is not directly validated. Adding data on nasal tissue temperature or citing similar studies could substantiate this claim.

**Response:** Thank you for the valuable comment. In the revised manuscript, we have cited a study that supports the effect of cooling in achieving hemostasis.

2.Bleeding Time Measurement: The criteria for determining complete hemostasis (absence of visible blood pooling) should be supported with additional references or validation.

**Response:** Thank you for the valuable comment. In the revised manuscript, we have supported the determination of hemostasis with a reference.

Results:
1.The variability in bleeding times (e.g., control group SD = 75.7 seconds) raises questions about potential inconsistencies in wound creation or animal responses. Addressing this variability in the discussion would be useful. (insert)

**Response:** We appreciate the insightful comment. Accordingly, we have discussed the variability in bleeding times in the revised manuscript.

2.The results rely solely on text and tables, which, while clear, lack complementary visual aids like graphs or charts.

**AUTHOR:** Thank you for your suggestion. Accordingly, two graphs have been included in the revised manuscript.

Discussion:
1. Indirect comparisons: The discussion summarizes the findings of other hemostatic agents but does not directly compare their outcomes (e.g., bleeding times, efficacy rates) to the Stop Nosebleeds device. It lacks quantitative comparisons, such as how the bleeding times achieved with ABS or MPH differ from those achieved with the Stop Nosebleeds device.

**RESPONSE:** Thank you for the suggestion. We have edited the paragraph to include the specific bleeding times related to ABS and MPH reported in the studies that allow a quantitative comparison to the Stop Nosebleeds device.

2.No focus on device uniqueness: While it mentions similarities to other devices, the unique advantages or disadvantages of the Stop Nosebleeds device (e.g., simplicity, cooling mechanism) compared to ABS, MPH, or oxidized cellulose are not highlighted.

**RESPONSE**: Thank you for the suggestion. In the revised manuscript, we have included a paragraph on the uniqueness of the Stop Nosebleeds device.

3.Contextualize for clinical use: Address why the Stop Nosebleeds device could be advantageous in real-world scenarios (e.g., first-aid use, portability, or safety in anticoagulated patients).

**RESPONSE**: We appreciate the valuable suggestion. These points have been included in the revised manuscript.

**Reviewer #3:**

Dear authors,
Thank you for this interesting manuscript about a new device to stop nasal bleeding. Although I think this manuscript may be interesting as initial research to keep developing the device, I found there are major thing that need to be addressed before publication.

General

English revision necessary

**Response:** English language editing was carried out while revising the manuscript.

No preoperative blood sampling? How do you know coagulation was normal? And platelet count or other variables that may affect bleeding? Although each animal was its own control, it may have affected results, please, state this in the text.

**Response:** The animals were sourced from a reputable supplier, where they were born and raised in a controlled, healthy environment. Routine blood work was conducted prior to the experiment including CBC and chemistry tests and they were clinically observed for any signs of abnormal bleeding or clotting, such as excessive bruising, prolonged bleeding from small cuts, or spontaneous bleeding. Epistaxis can be life threatening and the primary objective of this study was to evaluate the effectiveness of the Stop Nosebleeds device in controlling epistaxis, particularly in emergency situations, regardless of whether the patient has a coagulation disorder. Additionally, the device has been tested on heparinized animals, showing a significant reduction in bleeding duration compared to manual compression.

This information has been updated in the revised manuscript.

Major changes

Pg 7, lines 7-8. How did you ensure all punch defects were same depth and affecting same structures? It can massively change bleeding. Please, state this in the text.

**RESPONSE:** Thank you for the comment. The suggested information has been included in the revised manuscript as follows: A caliper was used to verify the consistency in size and depth of all punch defects. Additionally, the veterinary surgeon performing the punch defects is highly trained to apply the same technique and pressure with each procedure, ensuring uniformity in both size and depth.

Pg 7, line 15. How did you control time to haemostasis? Relieving the pressure periodically? How was this performed (both with manual compression and the device)? Please, state this in the text.

**Response:** Thank you for your valuable comments. We have clarified the above queries as follows in the revised manuscript:

Manual compression was always performed first on the right nostril (control group) to ensure consistency in the procedure. The left nostril (experimental group) was treated afterward using the Stop Nose Bleed Device. This sequential approach allowed us to compare the effectiveness of the device with standard manual compression while maintaining a controlled experimental setup. To minimize the potential influence of local hemostasis from the first intervention on the second, we ensured that an adequate time interval was maintained between procedures. Additionally, both nostrils were treated as independent sites since each had its own vascular supply, reducing the likelihood of crossover effects. Randomization was not applied in this setup to maintain methodological consistency and to ensure that manual compression, as the standard treatment, was always performed under controlled conditions first. This approach allowed for a clear comparison between interventions while preventing variability in external factors that could arise from alternating the order of treatments. For both, manual compression and the device, the pressure was gradually released to check for ongoing bleeding. Additionally, a second indication for hemostasis is the clot formation. When the clot is formed the pressure is released or the device is removed. After relieving pressure or removing the device, the animal is monitored for any signs of recurrent bleeding e.g. pooling blood or oozing.

Pg7, line 7. After induction, how was anaesthetia monitored?? How do you know arterial blood pressure and the other variables were normal and they did not affect bleeding? How did you control anaesthetic depth? Any preoperative analgesia administered? Please, state this in the text.

**Response:** After induction, a pulse oximetry was placed on the goat’s tongue to continuously measure the heart rate and the oxygen saturation of the blood. Additionally, physical indicators such as eye position, muscle tone, jaw tone, etc.; were used to assess the anesthesia depth. Blood pressure was not monitored during the procedure, only heart rate, body temperature, ECG and oxygen saturation and they were all normal throughout the procedure. Flunixin Melgumine 1.1 mg/kg was given pre-operatively via IV.

Pg 9. Isoflurane or sevoflurane were used? Change it in the text or figure as necessary.

**Response:** Sevoflurane was used. The change has been documented in the revised manuscript.

Pg 7, line 13. Manual compression was always performed first? How was controlled that local haemostasia was not affecting second bleeding?? Why not randomizing? Please, state this in the text.

**Response:** Manual compression was always performed first. The decision not to randomize the order was based on the goal of maintaining a consistent approach and controlling variables in a predictable and reproducible manner. This information has been included in the revised manuscript.

Pg 10, line 2. Your primary outcome was the number of wounds which stop bleeding. There is no information about this in the results section. If that was your primary outcome, why the sample size calculation was performed with time (following ARRIVE guidelines)? Please, state this in the text.

**Response**: We appreciate the reviewer comment. The primary and secondary outcomes are now clearly defined in the manuscript. Since the primary outcome is the bleeding time, so we estimated the sample size based on our primary outcome. This information has been included in the revised manuscript.

Pg 10, line 10. In the sample size calculation, you obtained 10 animals, but then, you performed 2 different experiments with only 5 animals per group, explain that (can we trust results of the two separate groups? Enough number of animals per group?). Where did you obtain your mean difference between groups to perform the sample size calculation? Previous studies? Preliminary study? Please, state this in the text.

**Response:** Thank you for your insightful comments. The procedure of sample size calculation is now clearly defined in the manuscript as requested. The sample size was determined based on the methodology outlined by Festing et al. (22), ensuring the study was adequately powered to detect meaningful differences and thus the number of animals per group was appropriate for statistical analysis. Regarding the mean difference used for the sample size calculation, it was defined based on a similar study conducted by Kurtaran et al. (1).

Pg 12, results. Redundant information, no need for text and table telling the same.

**Response:** We have edited this section to make it concise.

Pg 13, line 11. Antibiotic and anti-inflammatory administration should be moved to Materials & Methods.

**Response:** Thank you for the comment. As per the suggestion, this information has been removed from the discussion.

Pg 14, lines 4 - 6. How is your device compared to other methods? Advantages and disadvantages?

**Response:** Thank you for the suggestion. In the revised manuscript, we have discussed the unique advantages and disadvantages of the device compared to other methods.

Pg 14, lines 7 - 9. Paragraph about antibiotics / anti-inflammatory not necessary.
**Response:** Thank you for the comment. As per the suggestion, this information has been removed from the discussion.

Pg 14, lines 9 - 11. Haemostasis was achieved with both methods, please, state this in the text to make it clear.

**Response:** Thank you for the suggestion. We have clarified this in the revised manuscript.

Pg 14, lines 12 - 13. You cannot state that it may be highly effective in clinical settings, because, as you stated in the introduction, epistaxis can be caused by multiple situations and pathologies and you just performed a punch (lesion) please, rephrase and change this in the text to make it clear.

**Response:** Thank you for the comment. As per the reviewer’s suggestion, these lines have been rephrased in the revised manuscript.

Pg 14, discussion. How do we know that the pressure and cold from the device did not affect nasal mucosal integrity long-term? Any studies on that? Please, state this in the text.

**Response:** We have included this point as a suggestion for future studies.

Thank you very much for this interesting manuscript.

Your faithfully.

**Reviewer #4:**

The topic is quite interesting but the document is not in the format of the journal nor does it meet the minimum parameters of the journal.

Be careful with the line number. The abstract exceeds 300 words. There is no homogeneity throughout the document. It is very disorderly. The title of materials and methods is missing, as well as the guide for the protocol. The figures should be put “Fig.1”, “Fig.2” etc. The tables are not in the correct format. There is a lack of coherence in the writing. Only 20.6% are citations from 2019 to 2024 which means that 79.3% are sources under 5 years old. Citations number 26, 27 and 28 are not listed in the bibliography. All citations are not found with Vancouver standards, which is what the journal indicates, but a mixture of this with apa standards. Be careful, and also take into account the homogeneity in these same.

**Response:** Thank you for the comments. We have edited to abstract to meet the word limit of 300 words. Recent references have been included wherever possible. Other formatting suggestions have also been made.

**Reviewer #5:**

Abstract: Appropriate and crysp
Introduction: Appropriate and satisfactoryReview
Materials & Methods: Research techniques and methodology used are appropriate with standard statistical design application. Planning and conduct of experiments are appropriate as per the topic chosen.
Results & Appendices: The result have been appropriately presented in an elaborate and systemic manner, precisely interpreted and properly discussed with appropriate discussion.
Discussion: Discussion is critical with valid justification addressing all the objectives mentioned
Summary: Appropriate and satisfactory

**Reviewer #6: -**

Heparin used in control group and other with stop bleeding device and regular
- ways of conducting bleeding in experimental animal not clear

**Response:** Thank you for the comment. We have added more details regarding how bleeding was conducted in the experimental group.

- Statistical analysis can be modified with positive and control group with standard deviation

**Response:** We appreciate the suggestion and have accordingly added the new analysis in the revised manuscript.

- IACUC year agreement of protocol approval should be stated for experimental design

**Response:** Thank you for pointing that out. We have added the year of protocol approval in the revised manuscript.

- References need more up date from 2019 until now

**Response:** We have included more recent references wherever possible in the revised manuscript.

**Reviewer #7:**

In the abstract section, the method and results should have been given piece by piece, and should have been written without specifying them separately.

**Response:** Thank you for the comment. We have edited the abstract as suggested.

The photographs used in the material method section should be referenced if they are the photographs taken by the authors themselves.

**Response:** The figures belong to one of the co-authors in this study who is also the inventor of the Stop Nosebleeds device. Patent number 4538 from KACST, 10 Feb2015

“Tool to stop the nosebleed”, Shrouq aljanobi.

There are font differences in the article.

**Response:** The manuscript now maintains uniformity in the fond type.

If the right and left nasal mucosa of the same animal were to be used, the number of animals should have been higher, it is quite insufficient.

**Response:** The procedure of sample size calculation is now clearly defined in the manuscript as requested. The sample size was determined based on the methodology outlined by Festing et al., ensuring the study was adequately powered to detect meaningful differences and thus the number of animals per group was appropriate for statistical analysis.

Clinical parameters indicating the health status of the animals should have been clearly presented.

**Response:** The animals were sourced from a reputable supplier, where they were born and raised in a controlled, healthy environment. Routine blood work was conducted prior to the experiment including complete blood count and chemistry tests and they were clinically observed for any signs of abnormal bleeding or clotting, such as excessive bruising, prolonged bleeding from small cuts, or spontaneous bleeding. This information has been included in the revised manuscript.

The specified device is available for use in humans, Animal and human nasal mucosa and epithelium are histologically different. This difference may cause problems in comparing the results with other articles. Human structure and tissue are closer to laboratory animals, so why was a goat chosen, it would have been more appropriate to do it on a laboratory animal.

**Response:** Thank you for the comment. Domestic goats were chosen as the experimental animals because their nasal blood ﬂow most closely resemble that of human beings.

We have justified the use of goats with appropriate references in the revised introduction. Moreover, owing to the larger size of the Stop Nose device smaller animals, such as rabbits, would not have been suitable due to their smaller nasal passages.

.The reference articles used are old, the number of articles in the last 5 years is low.

**Response:** We have included more recent references in the revised manuscript.

**Reviewer #8:**

In the abstract and a large part of the manuscript it's not clear if the tested device is expected ultimately to be used in human or goat nasal bleeding.

**Response:** Thank you for the comment. This has been made clear in the revised manuscript.

In the abstract, it might be wise to briefly outline the mechanism of action of this device, the descrition of the method and the chronology of the acts are not clear,

**Response:** Thank you for the comment. This has been updated in the revised manuscript.

the conclusion of this study on goats seems to have been extrapolated to human without sufficient caution in the terms used.

**Response:** Thank you for the valuable comment. The conclusion section has been edited accordingly.

In the first part of the introduction it's not clear if the authors talk about goats or humans.

**Response**: We appreciate the reviewer’s insight. We have revised the introduction that clearly indicates when we are referring to humans and goats.

In the material and method section, the description of the device is not clear. An expansion of the device is mentionned but this is not described precisely enough.

**Response:** Thank you for the comment. We have revised the description of the device with greater clarity and references.

Even though it seems obvious, there is one or two references missing on the effect on temperature ans compression on the speed of clot formation and stopping of bleeding.

**Response:** Thank you for the comment. We have added appropriate references to support the sentences related to effect on temperature ans compression on the speed of clot formation.

A reference would also be useful to support the fact stated here that goat's nasal blood flow most closely resembles that of human beings.

**Response:** Thank you for the comment. This has similarity has been referenced accordingly in the revised manuscript.

On the other part the anatomy of goat's muzzle seems a little bit different from these of the human's nose, so the good coaptation of the device in either species might be questionable.

**Response:** We agree with the reviewer and have discussed this in the study limitations.

What is normal diet of the experimental animals during the study.

**Response:** They were fasted for 18 hours and water was withheld for 2 hours before anesthesia

Some informations about the experimental animals mentioned in the result part like age and weight would have a better place in the material and method section.

**Response:** Thank you for the suggestion. Accordingly, this information has been shifted to the methodology.

Description of anesthesy doesn't mention the use of premedication (which could influence the vasomotor status of the mucosa and so the bleeding). Is this the case ? The description and chronology of experimental acts as the road map of the protocol is not clear.

**Response:** Thank you for the comment. The suggested information has been included in the revised manuscript as follows: The animals were allowed to acclimatize for one week prior to anesthesia. They were fasted for 18 hours and water was withheld for 2 hours before anesthesia. Flunixin (1.1 mg/kg) was administered 30 minutes before anesthesia induction. Ketamine (10 mg/kg) and xylazine (0.2 mg/kg) were given via intramuscular injection, followed by atropine (0.15-0.3 mg/kg) via the same route. An intravenous catheter was then placed in the jugular vein. Anesthesia was induced with propofol (4 mg/kg) and then maintained with sevoflurane (2-3%). The animal was intubated and mechanically ventilated. Stomach tube was placed to decrease the risk of aspiration. Eye position, muscle tone and jaw tone were used to assess the depth of anesthesia. Heart rate, respiratory rate, body temperature, oxygen saturation were monitored throughout the procedure.

The lab profil mentioned as part of a data collection sheet should be described with more detail as the protocol of assessment of "re-bleeding" (timing of examination and criteria)

**Response:** Thank you for the comment. Accordingly, we have described the lab profile section with more details in the revised manuscript.

In table 1 goats which were heparinized should be identified.
In the context of One Health and good practices about antibiotic use is it really necessary to administer antibiotics for two 5mm diameter wounds surgicaly performed in the cranial part of the nasal mucosa. At any rate, this precision should be taken up in the material and methods rather then in the results section.

**Response:** Thank you for the comment. We have removed the information related to antibiotics from the discussion section.

Again in the discussion there is confusion between the condition, the results ant the conclusions in goats and humans.

**Response:** We have clarified this in the reviewed manuscript.

The other studies in animal models mentioned used mainly topical or general chemical agents to stop bleeding and seem to me difficult to compare with or to support the findings in the reviewed study about a mechanical device.

**Response:** We have added included studies using devices to stop epistaxis in the revised manuscript.

In the other hand, if we come back to the idea of carrying out this experiment on goats, we can see that most of the other animals models mainly concern rabbits.

**Response:** Domestic goats were chosen as the experimental animals because their nasal blood ﬂow most closely resemble that of human beings. We have justified the use of goats with appropriate references in the revised introduction. Moreover, owing to the larger size of the Stop Bleeding device smaller animals, such as rabbits, would not have been suitable due to their smaller nasal passages.
